# Design and synthesis of folate receptor-targeted squaraine dye complex for photodynamic therapy

**DOI:** 10.55730/1300-0527.3755

**Published:** 2025-08-23

**Authors:** Ömer SONKAYA

**Affiliations:** Department of Food Processing/Milk and Production Technology, Technical Sciences Vocational School, Aksaray University, Aksaray, Turkiye

**Keywords:** Squaraine dye, folate receptor, bioimaging, cancer, photodynamic therapy

## Abstract

In recent years, new treatment methods have been developed in addition to traditional treatments to eliminate cancer, one of the deadliest diseases. Given the shortcomings of conventional therapies, including the emergence of drug resistance and selectivity to the target cell, a considerable number of researchers have directed their attention toward the investigation of alternative, less invasive strategies for the treatment of cancer. Photodynamic therapy (PDT) has received great attention in the scientific community as it is seen as a promising method of cancer treatment. Various approaches have been adopted to identify new and more effective photosensitizers (PSs) that reduce adverse effects. In this context, it is suggested that squaraine dyes could potentially overcome the disadvantages associated with conventional PSs. Moreover, conjugating targeting agents such as folic acid (FA) to these PSs is suggested as a promising approach that can provide rational solutions for their applications in PDT.

In this study, the SQ-FA compound, obtained by conjugation of squaraine dye and folic acid (SQ-FA), was synthesized, its structure was characterized by various methods, and cytotoxicity studies were presented. SQ-FA is designed as a diagnostic and therapeutic tool for use in cancer treatment for human lung carcinoma epithelial cells (A549). In addition, biocompatibility studies of dermal fibroblast cell lines (L929) were carried out. SQ-FA can function as a PS and be used as a targeting agent. Spectroscopic analysis and optical properties of SQ-FA, a new dye belonging to the squaraine family, were investigated.

## 1. Introduction

Cancer is a fatal disease that arises from the uncontrolled division, proliferation, and growth of cells in different parts of the body. Since cancer is a disease with a high mortality rate and the mechanism of its occurrence in humans is not fully elucidated, its treatment remains highly challenging. Although conventional treatment strategies have not been able to cure cancer, photodynamic therapy has recently gained increasing attention [1]. The fact that traditional treatments have serious side effects and are not very effective on metastatic tumors has opened a new field for drug designs with high therapeutic effectiveness and low side effects in cancer treatment [2,3].

Cancer is the most common disease worldwide, with 9.7 million deaths in 2022 [4]. Its prevalence and mortality are increasing for a variety of reasons, including population aging and growth, and social and economic causes (e.g., hereditary, poor diet, air pollution, stress, smoking, and alcohol use). It is estimated that 20% of men and 17% of women will get cancer in 2022, and unfortunately, about 10% of men and 8% of women who get cancer will die from it [4]. Although research has been conducted to improve existing treatment methods for cancer, there have been no significant changes in cancer detection and treatment. Unfortunately, none of the imaging techniques can be carried into the operating room due to tissue manipulation and patient compliance [5–7]. Therefore, a real change in surgery is essential [8]. Additionally, although there have been advances in imaging techniques, standard procedures are limited in distinguishing healthy tissue from malignant lesions [9,10]. As a result, new solutions are needed to diagnose and treat tumors [11].

Over the last decade, new treatment modalities have been reported alongside conventional therapies to eradicate cancer, one of the deadliest diseases [12]. In light of the shortcomings of conventional therapies, including the emergence of drug resistance, poor selectivity to the target site, and low efficacy, numerous researchers have dedicated their efforts to investigating alternative, less invasive strategies for combating cancer [13,14]. In recent times, photodynamic therapy has come to the fore as a promising avenue in the clinical management of cancer, offering significant potential for advancement [15,16].

Unlike traditional treatment methods in cancer treatment, photodynamic therapy (PDT) is a promising method for therapeutic [17] and antimicrobial applications [18] thanks to its high selectivity [19] and low-invasive nature [19–21]. In PDT treatment, a photosensitizer (PS), light with appropriate energy, and oxygen are essential [22,23]. However, when a PS is activated by low-energy light at the optimum excitation wavelength in an oxygen-rich environment, it produces reactive oxygen species (ROS) that lead to oxidative damage that triggers the death of cancer cells; this process occurs through necrosis and/or apoptosis [24].

In recent years, the PDT activity of many different molecules, including some natural products, has been examined, and its potential is high. In particular, synthetic dyes have been studied for anticancer activity [25,26]. Squaraine dyes have the potential for use in PDT. It also provides good absorption in the near-infrared (NIR) region between 600 and 800 nm. It enables a high signal-to-noise ratio for biomedical applications and bioimaging [27,28]. Furthermore, PSs should exhibit optimal absorption between 600 and 800 nm to guarantee robust light penetration into tissues. Consequently, agents that demonstrate robust absorbance and fluorescence in the NIR region, such as squaraine, are more conducive to photodynamic therapy (PDT) treatments [29].

Since their discovery in 1965 [30], squaraine dyes have been extensively studied by scientists for applications in organic solar cells and sensors [31–33]. Recently, bioimaging and photodynamic therapy activities have begun to be investigated. In contrast, investigations into their relevance and applications in the bioimaging field were significantly fewer. Squaraine dyes are a class of dyes that are derived from the compound known as squaric acid. They are characterized by their square-shaped core structure and optical properties. These properties make squaraine dyes useful in a range of applications [34], including fluorescent labeling, sensors [35], and photovoltaic devices. Their distinctive structure and optical properties [36] make squaraine dyes valuable tools for researchers and scientists working in fields that require precise control over light absorption and emission properties [37].

Folate, also known as folic acid (FA), is important for the biosynthesis of nucleotide bases in the eukaryotic cell [37]. Folate receptors are frequently expressed on cancer cell surfaces. Due to the high binding coefficient of FA to excess folate receptors on the cell surface, the use of folate-targeted agents has emerged as an attractive method for the treatment of many cancers [38,39]. Detectable amounts of folate can generally only be detected in the membrane of certain cancer cells and some macrophages [40–48]. FA is easier to obtain and more useful for detecting cancer cells than other targeting agents [49,50]. Moreover, to date, very few FA agents that actively target cancer cells have been designed in biochemistry and medicine [51–54]. The incorporation of FA is of particular importance because it enables the versatility of ligand screening using different targeting molecules for other potential applications. Moreover, a strategy that involves preloading bioactive drug payloads into the liposome core can be employed, thereby minimizing undesirable side reactions [37].

**Figure 1:**
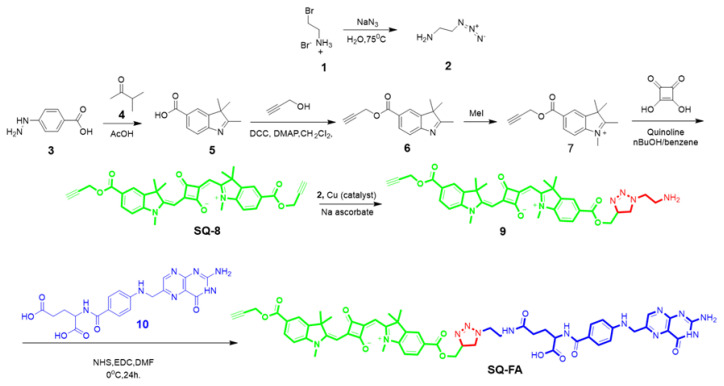
Synthesis of SQ-FA.

Herein, the SQ-FA compound was synthesized as a result of the conjugation of squaraine dye, which can function as a PS, and FA, which can be used as a targeting agent (Figure 1). PS with SQ-FA was designed as a diagnostic and treatment tool for use in cancer treatment.

## 2. Materials and methods

### 2.1. General considerations

Chemicals were used directly from commercial suppliers. A549 and L929 cell lines were obtained from Kirikkale University Scientific and Application Research Center. Cell imaging experiments were performed with a Leica DM IL LED inverted fluorescence microscope. The melting point was determined using the electrothermal model IA9100. Nuclear magnetic resonance (NMR) spectroscopy was performed using an Agilent Premium Compact 600 MHz NMR ( Agilent Scientific Instruments, USA) instrument at the Central Research Laboratory of Çankırı Karatekin University. Mass spectra were recorded on three different instruments: Thermo Scientific TSQ Quantum Access MAX (Thermo Fisher Scientific – USA), Shimadzu GC-MS Q2010 Direct Injects (Shimadzu Exellence in Science Japan), and Bruker Microflex LT MALDI-TOF MS (Bruker,USA). Ultraviolet–visible (UV–Vis) absorption spectra were recorded using a Lambda 35 spectrophotometer (PerkinElmer, USA).

Compounds 5–7 and SQ-8 [55] were synthesized according to previously reported methods. The synthesis was performed by adapting the procedures described for compound 2 [56] and SQ-FA [57] in the literature.

### 2.2. Synthesis of 2-aminoethyl-1-amine

Commercially available 2-bromoethylaminohydrobromide (5.0 g) and NaN_3_ (4.76 g) were dissolved separately in 5 mL of water and mixed with a magnetic stirrer. Refluxing was then carried out with the Milestone microwave synthesis system (Milestone Helping Chemists, Italy) at 800 W and 80 °C for 2 h. After cooling to 25 °C, diethyl ether (50 mL) was added to the reaction mixture, and work-up was performed. The organic fraction was removed using a separatory funnel. The solution was then dried using anhydrous sodium sulfate. Subsequently, the light yellow oil fraction (2.1 g) was stored in the refrigerator at −20 °C for 1 day and then decanted. The solvent was removed from the resulting solid. The product is liquid at room temperature. The experiment produced an 85% yield. ^1^H NMR (600 MHz, CDCl_3_) δ 3.33 (t, 2H), 2.84 (t, 2H), 1.57 (s, 2H). ^13^C NMR (151 MHz, CDCl_3_) δ 41.28 and 54.58 ppm.

### 2.3. Synthesis of compound 9

Compound SQ-8 (300 mg) and compound 2 (52 mg) were added to 10 mL of N,N-dimethylformamide (DMF). CuSO_4_.5H_2_O (13 mg) and sodium ascorbate (20 mg) were dissolved separately in water (5 mL) and added slowly with a syringe simultaneously. The reaction was carried out in an inert atmosphere at room temperature for 24 h. The resulting suspension was centrifuged, and the pale yellow supernatant was decanted. After several washes with water, the solid was dried under vacuum to give 282 mg of compound 9 in 82% yield.^1^H NMR (600 MHz, CDCl_3_) δ (ppm): 8.07(m, 2H), 8.00 (m, 2H), 7.79 (s, 2H), 7.00 (bs, 2H), 6.00 (s, 2H), 5.47 (s, 2H), 4.93 (s, 2H), 4.40 (s, 2H), 3.55 (s, 2H), 3.46 (s, 2H), 3.22 (s, 2H), 2.52 (s, 1H), 2.25 (s, 1H), 1.78 (m, 8H), 1.56 (bs, 4H), 1.42 (s, 2H), 1.24 (s, 2H).^13^C NMR (151 MHz, CDCl_3_) δ (ppm): 165.98, 165.29, 161.82, 143.08, 142.79, 141.96, 135.63, 130.84, 130.70, 125.45, 125.34, 124.70, 123.75, 119.26, 115.99, 115.49, 108.82, 96.04, 88.59, 77.60, 75.03, 65.74, 58.06, 53.49, 52.53, 48.95, 41.87, 30.29, 29.70, 27.06, 24.13, 15.13, 14.08.

### 2.4. Synthesis of SQ-FA

100 mg of commercially available FA in 10 mL of DMF was sonicated with an ultrasonic water bath. It was then allowed to cool by creating a salt-ice bath environment. N-hydroxysuccinimide (NHS) (26 mg) and EDC (44 mg) were dissolved in DMF and added to the reaction medium, and the resulting mixture was stirred in an ice bath for 30 min. Compound 9 (153 mg) dissolved in 2.5 mL of DMF was added to the reaction medium with a syringe and stirred at room temperature for 24 h. It was precipitated with acetone and dried under vacuum to give a blue solid in 12% yield. The melting temperature was controlled and 360 ^o^C decomposition occurred. ^1^H NMR (600 MHz, dimethyl sulfoxide [DMSO]-d_6_): 11.37 (s, 1H), 10.50 (s, 1H), 9.02 (s, 1H), 8.61 (s, 1H), 8.26 (s, 1H), 8.14 (d, 1H), 8.07 (d, 1H), 7.99 (s, 1H), 7.69 (s, 1H), 7.61 (s, 1H), 6.65–6.62 (m, 2H), 5.94 (m, 1H), 5.84 (m, 1H), 5.69 (m, 1H), 4.92 (m, 2H), 4.55 (m, 2H), 4.45 (m, 2H), 4.30 (m, 1H), 3.58 (m, 2H), 3.13 (t, 1H), 3.00 (m, 2H), 2.82 (m, 6H), 2.71(s, 2H), 2.56 (m, 3H), 2.31 (m, 2H), 1.92–1.90 (m, 2H), 1.67 (m, 6H), 1.20 (s, 3H), 0.96 (t, 3H) [57].

### 2.5. Live cell imaging

SQ-FA cell images were produced using the A549 cell line with a Leica (Leica Microsystems, Germany) microscope. Imaging was performed by excitation with a 635 nm laser. A549 cells were grown using Dulbecco’s modified Eagle medium (Sigma-Aldrich, USA) (DMEM) at 37 °C in a 5% CO_2_ atmosphere. After this step, the cells were incubated for 1 h and washed three times with phosphate-buffered saline (PBS). Finally, the cells were incubated with Hoechst nuclear stain for 15 min.

### 2.6. Biocompatibility of SQ-FA

This study used the cell culture method for the preliminary evaluation of SQ-FA biocompatibility. The L929 mouse fibroblast cell line was selected as being considered a normal fibroblast, is easy to cultivate, and has been used as a reference cell line in toxicity tests. L929 cells were plated in DMEM at 37 °C in a 5% CO_2_ atmosphere. After 24 h, the cells were moved to 96-well plates at concentrations ranging from 0.015 to 0.5 mg/mL and incubated overnight. The next day, the cells were washed three times with PBS. Ten microliters of 3-(4,5-dimethylthiazol-2-yl)-2,5-diphenyltetrazolium bromide (MTT) solution was added to each well for viability analysis. The cell status was then recorded at 570 nm with a BMG Labtech microplate reader.

### 2.7. (Photo)cytotoxicity and MTT assay

The MTT assay was utilized to ascertain the level of toxicity exhibited by SQ-8 and SQ-FA. A viability percentage below 70% in the sample is generally considered an indicator of potential toxicity. A549 cells were prepared as a suspension in 96-well culture plates at a density of 1.0 × 10^4^ cells per well and incubated for 24 h. The extraction process was continued at 37 °C for 24 h in accordance with the International Organization for Standardization (ISO) 10993-12 standard. During this process, the extraction solution was prepared at a concentration of 0.2 g/mL and then filtered. The prepared extracts were then subjected to an incubation process at 37 °C, utilizing a series of seven distinct concentrations ranging from 0.015 mg/mL to 1.0 mg/mL. It is noteworthy that the concentration of 1.0 mg/mL represented 100% of the sample in this experiment. Each sample was analyzed with six replicates. Positive and negative controls were performed in triplicate. A polyurethane film containing 0.1% zinc diethyldithiocarbamate (ZDEC) was used as a positive control, while a high-density polyethylene film was used as a negative control. The medium was used as a single blank. Following a 24-h period of incubation, the medium from the wells was simply removed, and 50 μL of MTT solution (1 mg/mL) was added to each well. Following a further 2 h of incubation at 37 °C, 100 μL of isopropanol was added to the wells. The final step involved measuring the absorbances of the 96-well plates at 570 nm using a microplate reader. This measurement was carried out to determine cell viability. The percentage of viability was then calculated and compared with the control group.

### 2.8. Fluorescence quantum yield

The fluorescence quantum yield (Φ_FL_) of SQ-FA was calculated by excitation at 620 nm according to the method given in the literature [58].

### 2.9. ^1^O_2_ quantum yield

We calculated the ^1^O_2_ quantum yield (Φ_Δ_) of SQ-FA using methylene blue (MB) as the 412 nm standard. The method reported in the literature was used exactly as described [59].

## 3. Results

**Figure 2:**
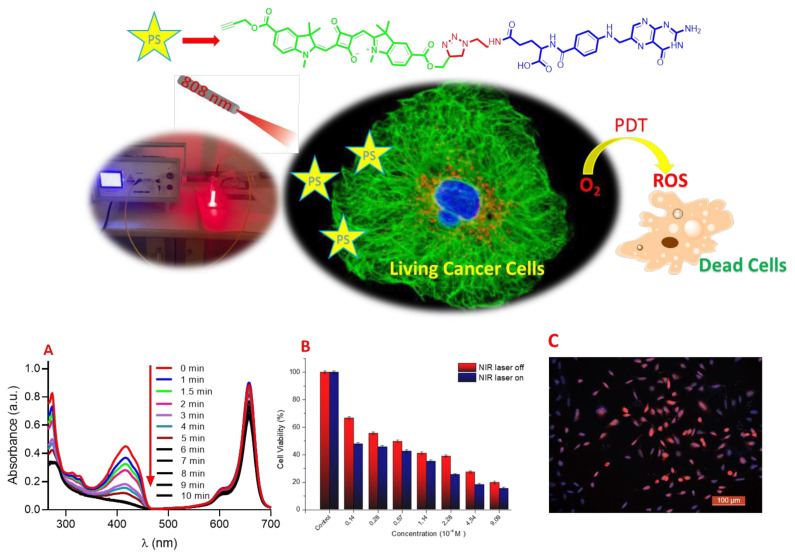
Schematic representation of applications of conjugated squaraine dye SQ-FA.

In this study, which is schematically shown in Figure 2, the rational design of a generic platform that can provide access to theranostics was carried out. In this context, the synthesis and characterization of a fluorescence-based compound that will enable cancer treatment has been carried out. In the design, on the one hand, a unit (FA) that can be used as a cancer-targeting agent; on the other hand, SQ-FA, a fluorescent dye suitable for bioimaging and PDT with its superior properties, was synthesized. The structures of the synthesized fluorescent dyes were elucidated by spectroscopic analysis, and their potential as photosensitizing agents was determined. The structure of SQ-FA was confirmed by analysis of NMR, Fourier-transform infrared spectroscopy (FTIR), and mass spectra.

**Figure 3:**
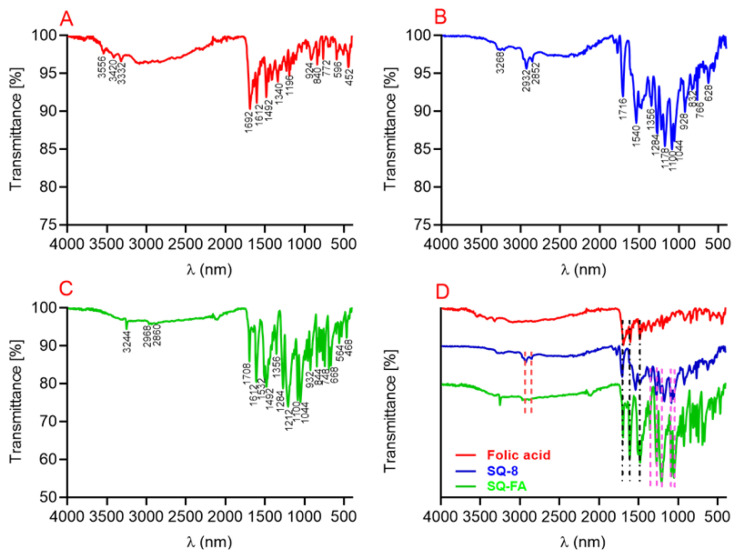
FTIR spectra of A) folic acid, B) SQ-8, C) SQ-FA, and D) merge.

When the FTIR spectrum (Figure 3) for compound SQ-FA is examined, the red spectrum belongs to FA (Figure 3A), the blue spectrum to compound SQ-8 (Figure 3B), and the green spectrum to compound SQ-FA (Figure 3C). When the spectra were examined comparatively, the vibrational bands corresponding to 2931 and 2866 cm^−1^ were attributed to aromatic hydrocarbons, whereas the band at 3250 cm^−1^ was attributed to the −OH group. Furthermore, the symmetric stretching bands of aliphatic hydrocarbons were observed at 2931 cm^−1^, 1660 cm^−1^ (C=O), 1386 cm^−1^ (COO-stretching), and 1092 cm^−1^ (C-NH_2_). In the reference spectrum of FA, the band corresponding to the −OH group became evident at 3250 cm^−1^. The band at 1696 cm^−1^ corresponds to the C=O stretching band of the carboxyl group, whereas the band at 1609 cm^−1^ corresponds to the −NH group. Furthermore, the specific band at 1480 cm^−1^ was attributed to the phenyl ring.

**Figure 4:**
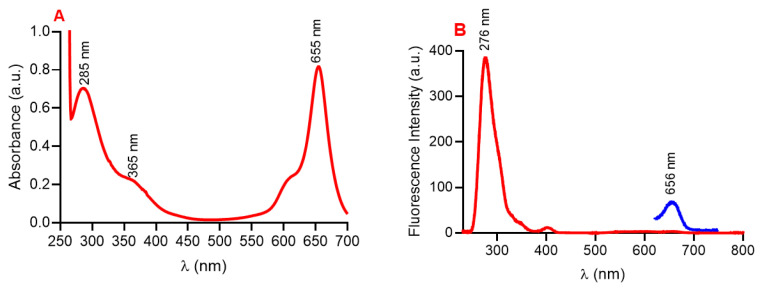
A) UV–Vis absorption, B) fluorescence spectra of SQ-FA (1.66 × 10^−6^ M) in DMSO (λ_ex_= 230 and 620 nm).

Following synthesis and structural characterization, an investigation was conducted into the photophysical properties of SQ-FA. The UV–Vis absorption spectrum of SQ-FA (1.66 × 10^−6^ M) in DMSO exhibited prominent signals of FA at 285 nm and 365 nm, while the signals of squaraine displayed a peak at 656 nm. (Figure 4A). Examination of the fluorescence spectrum revealed that the SQ-FA compound emitted at 276 nm and 683 nm when excited at 230 nm and 620 nm, respectively (Figure 4B). Using methods and calculations in the literature, the fluorescence quantum yield (Φ_FL_) of SQ-FA was determined to be 0.30 [58].

**Figure 5:**
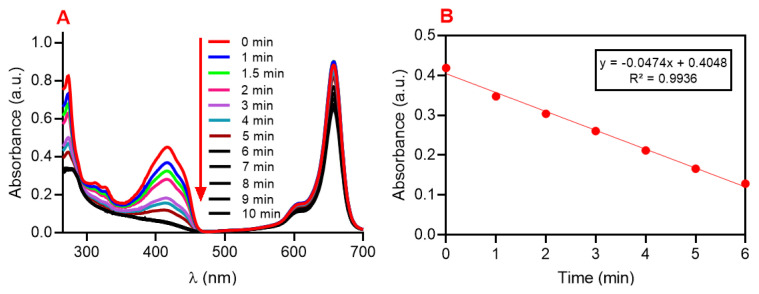
A) Generation of ROS upon irradiation of SQ-FA (15.40 x10^−3^ mg/mL, 655 nm) in the presence of DPBF (7.70 x10^−3^ mg/mL, 415 nm) in 0.01 M PBS (containing 1% DMSO, pH 7.4) with 630 nm laser photoirradiation at 1 W/cm^2^. B) Time-dependent bleaching of DPBF during irradiation of SQ-FA in PBS (containing 1% DMSO, pH 7.4).

The primary function of SQ-FA as a photosensitizing agent was evaluated using chemical trapping experiments. The 1,3-diphenylisobenzofuran (DPBF) trap molecule, one of the ROS trap molecules reported in the literature, was used to effectively capture the ROS produced during the investigation of the PDT efficacy of the SQ-FA compounds. Upon generation of ROS by the photosensitizer, a chemical reaction with DPBF occurs. Changes in the UV–Vis absorption spectrum of DPBF at 415 nm were monitored to determine whether the compound had PDT activity. For this purpose, DPBF was used as a chemical trap molecule to test the reactive oxygen generation capacity of SQ-FA. Figure 5 presents the results of the experimental study on photosensitization using SQ-FA. It is noteworthy that SQ-FA can generate significant amounts of ROS when exposed to red light in PBS (containing 1% DMSO, pH 7.4) (Figures 5A and 5B). In studies conducted using methylene blue (MB) as a reference, the quantum yield (Φ_Δ_) of ROS formation in the presence of PBS was found to be 1.69 [59].

The quantum efficiency of ROS production (Φ_Δ_) was compared between squaraine dye (SQ-8) and SQ-FA. The quantum efficiency (Φ_Δ_) of SQ-8 for ROS production was calculated to be 0.13 using methylene blue (MB) as a standard, whereas that of SQ-FA was calculated to be 1.69. (For MB, Φ_Δ_ = 0.52). It has been shown that FA in the structure of SQ-FA positively affects the quantum yield of ROS production (Φ_Δ_), which is higher than that of SQ-8 [39,55].

**Figure 6:**
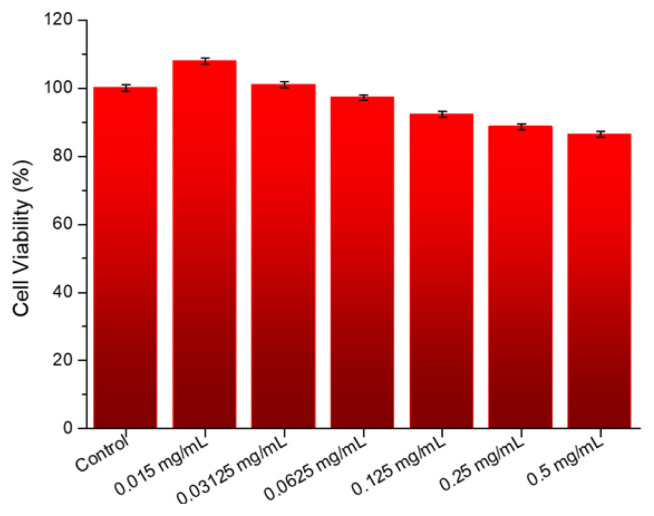
Viability of L929 cells after 24 h of incubation with SQ-FA at various concentrations.

In view of these encouraging results, we decided to test the hypothesis that SQ-FA is a potential candidate for in vitro studies. To this end, studies were first conducted with the L929 cell line, which is considered a normal fibroblast, and then with the A549 cancer cell line. Initial in vitro biocompatibility experiments were performed using L929 cells and the MTT assay to assess the biological safety of the material under investigation (SQ-FA). The cell culture method was selected as a preliminary tool for assessing the biocompatibility of SQ-FA. The L929 cell line, which is considered a normal fibroblast, was selected for use in this study because of its ease of cultivation and status as a reference cell line for evaluating cellular responses to chemicals. As shown in Figure 6, SQ-FA demonstrated no significant toxicity against L929 cells at concentrations ranging from 0.015 to 0.5 mg/mL, even after 24 h of incubation. The results demonstrated that SQ-FA exerted no cytotoxic effect on the L929 cell line, exhibiting a viability of over 86% even at elevated concentrations (Figure 6).

**Figure 7:**
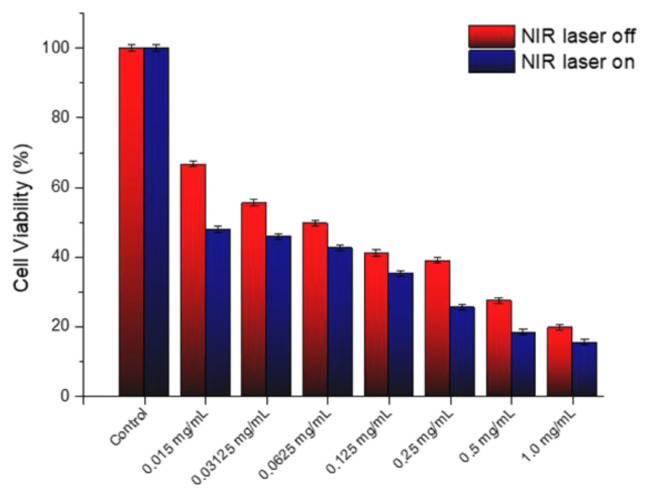
Viability of A549 cells incubated with SQ-FA for 48 h with and without 630 nm laser photoirradiation at 1 W/cm^2^ for 8 min.

A decline in cell viability of less than 70% compared to that of the control group may be designated as cytotoxic. As shown in Figure 6, the study conducted with the SQ-FA compound revealed that cell viability ranged from 86.9% to 110%. This finding indicates that SQ-FA formulations are biocompatible, and their phototherapeutic efficacy is a promising avenue for further investigation. An additional experiment was conducted to determine and evaluate the antiproliferative effects of SQ-FA on A549 cells. The in vitro antiproliferative activity of SQ-FA was investigated using A549 cells. The potential benefit of SQ-FA in photothermal therapy was demonstrated by treating A549 cells with SQ-FA at concentrations ranging from 0.015 to 0.5 mg/mL. The cells were then exposed to NIR laser irradiation (1 W/cm^2^, 630 nm) for 8 min. The results of the experiments are shown in Figure 7.

As shown in Figure 7, the combination of samples and laser irradiation exhibited increased efficacy in eradicating A549 cells at all concentrations tested. When NIR irradiation was present, cell viability varied between 15.42% and 47.83%, whereas in the absence of NIR irradiation, cell viability ranged between 19.73% and 66.65%. SQ-FA demonstrated a substantial antiproliferative effect, even at comparatively low concentrations (0.015 mg/mL), both in the absence and presence of NIR irradiation. This significant effect is due to a process that facilitates the conversion of absorbed light energy into molecular oxygen ROS in malignant tissues. Furthermore, Figure 7 shows a dose-dependent decrease in viability in A549 cells treated with SQ-FA, regardless of NIR laser irradiation. The figure demonstrates a clear relationship between increasing the SQ-FA concentration and decreasing cell viability. The observed dose-dependent toxicity suggests that it may be effective in the presence of photosensitizer drugs and may be beneficial in reducing potential side effects and nonspecific toxicity. Consequently, these findings indicate that the synthesis of SQ-FA with enhanced antiproliferative activity and negligible toxicity may represent a promising prospect for future chemotherapy applications.

**Figure 8:**
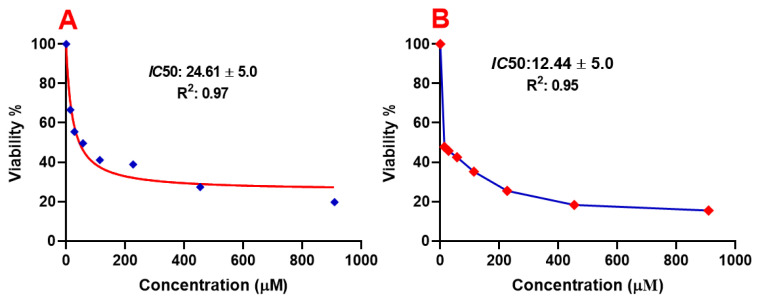
SQ-FA cytotoxicity effect against A549 cell lines (IC50 values), A) without irradiation and B) with irradiation.

In order to evaluate the cytotoxic effects of SQ-FA, the half-maximal inhibitory concentration (*IC*50) with and without irradiation was calculated using GraphPad Prism 8 software. The determined *IC*50 values corresponding to the dye concentration producing a 50% reduction in maximum cell viability are presented in Figure 8. It is noteworthy that the *IC*50 values calculated for the dye SQ-FA exhibited a cytotoxic effect of 24.61 μg/mL in the A549 cell line when examined in the absence of light (Figure 8A), whereas in the presence of light (Figure 8B), it was 12.44 μg/mL. The low IC50 value of SQ-FA can be attributed to the high ^1^O_2_ quantum yield and efficient mitochondria-specific ROS production upon light irradiation. This finding indicates that SQ-FA exhibits a high degree of selectivity for NIR-induced cellular toxicity.

**Figure 9:**
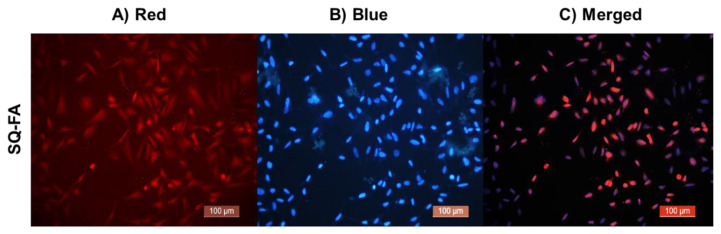
Fluorescence images of A549 cells incubated with A) SQ-FA (red), B) Hoechst (blue), and C) overlay of Hoechst and SQ-FA (merged). Scale bar = 100 μm.

Owing to its low toxicity, SQ-FA is considered a suitable option for live cell imaging. In this study, we investigated the cellular internalization of SQ-FA by A549 cells. Imaging was performed using a Leica microscope to observe the effects of SQ-FA on A549 cells. Figure 9 presents the results of this investigation in the form of fluorescence microscopy images of A549 cells after incubation with SQ-FA. The figure presents the combined images obtained by superimposing SQ-FA (red), Hoechst (blue), and both. This study demonstrated the potential of SQ-FA as an effective fluorescence imaging agent in living cells.

## 4. Discussion

In summary, the primary aim of this study is to target a cancerous cell with the assistance of FA and to eradicate the cancerous cell through the application of photodynamic therapy with the aid of photosensitizing squaraine dye. To this end, FA is employed as a targeting agent for cancer cells. The synthesis of SQ-FA dye, a new member of the squaraine family, was completed, and its characterization, photophysical properties, bioimaging capacity, and cytotoxicity were subsequently investigated.

The suitability of SQ-FA as a photosensitizer was assessed through the utilization of chemical capture assays. The quantum yield of ROS production (Φ_Δ_) of SQ-FA was found to be higher than that of SQ-8. Furthermore, bioimaging studies conducted on the A549 cell line demonstrated that it is an effective imaging agent, and cytotoxicity tests indicated that it can be a viable therapeutic agent when stimulated with or without a laser. In conclusion, the synthesized SQ-FA compound shows promise for the treatment of cancer cells, as it has the potential to form a stable ligand-drug complex for PDT.
